# Inactivation of Uropathogenic *Escherichia coli* in Ground Chicken Meat Using High Pressure Processing and Gamma Radiation, and in Purge and Chicken Meat Surfaces by Ultraviolet Light

**DOI:** 10.3389/fmicb.2016.00413

**Published:** 2016-04-14

**Authors:** Christopher H. Sommers, O. J. Scullen, Shiowshuh Sheen

**Affiliations:** Eastern Regional Research Center, United States Department of Agriculture, Agricultural Research Service, WyndmoorPA, USA

**Keywords:** UPEC, high pressure processing, gamma radiation, ultraviolet light, chicken

## Abstract

Extraintestinal pathogenic *Escherichia coli*, including uropathogenic *E. coli* (UPEC), are common contaminants in poultry meat and may cause urinary tract infections after colonization of the gastrointestinal tract and transfer of contaminated feces to the urethra. Three non-thermal processing technologies used to improve the safety and shelf-life of both human and pet foods include high pressure processing (HPP), ionizing (gamma) radiation (GR), and ultraviolet light (UV-C). Multi-isolate cocktails of UPEC were inoculated into ground chicken which was then treated with HPP (4°C, 0–25 min) at 300, 400, or 500 MPa. HPP D_10_, the processing conditions needed to inactivate 1 log of UPEC, was 30.6, 8.37, and 4.43 min at 300, 400, and 500 MPa, respectively. When the UPEC was inoculated into ground chicken and gamma irradiated (4 and -20°C) the GR D_10_ were 0.28 and 0.36 kGy, respectively. The UV-C D_10_ of UPEC in chicken suspended in exudate and placed on stainless steel and plastic food contact surfaces ranged from 11.4 to 12.9 mJ/cm^2^. UV-C inactivated ca. 0.6 log of UPEC on chicken breast meat. These results indicate that existing non-thermal processing technologies such as HPP, GR, and UV-C can significantly reduce UPEC levels in poultry meat or exudate and provide safer poultry products for at-risk consumers.

## Introduction

*Escherichia coli* are classified as commensal (natural microflora), or variants that cause disease such as intestinal pathogenic *E. coli* (iPEC) or extraintestinal (ExPEC) types. Groups of ExPEC include Neonatal Meningococcal *E. coli* (NMEC), Avian Pathogenic *E. coli*, (APEC), Sepsis-associated Pathogenic *E. coli* (SEPEC) and Uropathogenic *E. coli* (UPEC) (Mitchell et al., 2015). *E. coli* such as ExPEC (UPEC) are responsible for 75–95% of urinary tract infections (UTI) and uncomplicated cystitis and pyelonephritis (Nordstom et al., 2013). Fifty percent of women will contract one UTI in their lifetime, and 25% will have a recurrent UTI (Minardi et al., 2011; Bao et al., 2014). The number of UTI in the US is ca. 6–8 million annually, with ca. 100, 000 hospitalizations, ca. 23,000 deaths, and a health care burden of ca. $3.5 billion (Nordstom et al., 2013). The mechanism for contraction of a UTI is transfer of contaminated feces from the gastrointestinal tract to the urethra, and isolates associated with UTI invariably match the individual’s fecal microflora (Moreno et al., 2008).

The idea that extraintestinal foodborne pathogens such as the ExPEC might be responsible for UTI in humans is relatively new, and it has long been suspected they may be associated with illness outbreaks (Markland et al., 2015). The presence of ExPEC in poultry meat has been firmly established (Johnson et al., 2005; Mitchell et al., 2015). Studies have compared ExPEC isolates from food animals, food, and those from women with UTI and the incidence of ExPEC in poultry meat and have demonstrated both genetic similarity and identity between ExPEC from animals and food with those from humans with UTI (Cortes et al., 2010; Jakobsen et al., 2010a,b, 2012; Vincent et al., 2010; Bergeron et al., 2012; Mora et al., 2013). More importantly ExPEC isolated from animals and food can cause UTI in mouse model systems (Jakobsen et al., 2012).

Three non-thermal intervention technologies of interest to the meat and poultry processing industry, which are used commercially to improve food safety and extend shelf life, include high pressure processing (HPP), ionizing (gamma) radiation (GR) and ultraviolet light (UV-C) (Salvage, 2014). HPP subjects food to an elevated pressure of 100–1000 MPa typically at temperatures below 60°C. The mechanism by which HPP inactivates foodborne pathogens includes cell membrane and structure damage, ribosome dissociation, dissociation of DNA, and enzyme inactivation (Campus, 2010; Simonin et al., 2012). GR inactivates microorganisms by damaging their DNA indirectly through radiolysis of water and induction of oxidative damage or direct damage through breakage of the phosphodiester backbone in addition to oxidative damage to proteins and cell membranes (Taub et al., 1979; Diehl, 1995). UV-C kills microorganisms through induction of cyclobutane pyrimidine dimmers and 6-4 photoproducts in addition to protein damage (Krisko and Radman, 2010; Rastogi et al., 2010).

The purpose of this study was to determine the HPP and GR inactivation kinetics for ExPEC (UPEC) inoculated in ground chicken as well as the UV-C inactivation kinetics on poultry meat surfaces and in chicken purge on food contact surfaces. To the authors knowledge this is the first study to examine the inactivation kinetics of ExPEC in a food system.

## Materials and Methods

### Chicken

Ground chicken (92% lean) was freshly prepared and purchased at a local wholesaler (Lansdale, PA, USA) and evenly portioned into 90 g aliquots in polynylon pouches (Uline, Inc., Philadelphia, PA, USA), vacuum sealed to 50 millibars using a Multi-Vac A300 packager (Multi-Vac Inc., Kansas City, MO, USA) and then frozen (-70°C). The ground chicken was tested for presence of *E. coli* as described below and it was <1 CFU/g. Multiple chicken lots were tested and one with low *E. coli* levels was selected. Boneless skinless chicken breast and chicken skin was obtained fresh from a local butcher. Chicken purge was obtained from a local poultry processor and frozen (-70°C) until ready for use.

### *E. coli* Isolates

The *E. coli* isolates were obtained from the American Type Culture Collection (Manassas, VA, USA). These include 700414, 700415, 700416, 700417, 700336, and BAA-1161 (http://www.atcc.org), which were isolated from women with UTI. Multi-isolate cocktails of the pathogens were used as recommended for appropriate validation of non-thermal processing technologies (National Advisory Committee on Microbiological Criteria for Food [Nacmcf], 2006). The individual isolates were prescreened for resistance to HPP, GR and UV prior to use, and the D_10_ were consistent with results for our previous studies with iPEC (Sheen et al., 2015; Sommers et al., 2015; Sommers et al., unpublished data).

### *E. coli* Growth and Inoculation

The *E. coli* were cultured independently in 20 ml Tryptic Soy Broth (TSB) without dextrose to avoid development of acid resistance (BD-Difco, Sparks, MD, USA) using 50 ml sterile tubes at 37°C (150 rpm) for 18–24 h using a New Brunswick Model G34 Environmental Shaker (New Brunswick, Edison, NJ, USA). The bacteria were then sedimented by centrifugation (1,200 × *g*, Hermle Model Z206A, Hermle Labortechnik, Germany) and resuspended as a cocktail in 20 ml sterile 0.1% peptone water (SPW, BD-Difco).

Thawed ground chicken (10 g) was aliquoted into 2 oz. Nasco (Ft. Atkinson, WI, USA) Whirl-Pak bags, inoculated with 0.1 ml of UPEC, mixed manually for 1 min, and then sealed using the Multi-Vac A300 Packager. The final concentration of UPEC in the ground chicken was ca. 8–9 log CFU/g. The sample bags were then sealed in a second bag and stored at 4°C until HPP treatment or gamma radiation (ca. 2 h).

### High Pressure Processing Treatment

High pressure processing was performed using a laboratory scale pressure unit (Mini Food lab FPG5620, Stansted Fluid Power Ltd., Essex, UK), comprised of a double-jacketed thick-wall stainless steel cylinder (approximate volume of 0.3 L) having an internal stainless steel sample holder of 25.4 mm × 254 mm (diameter × length). The thick-wall cylinder was maintained at a set-point temperature in which heat transfer fluid continuously circulated from a refrigerated liquid chiller (Proline RP 855, Lauda, Germany). The pressure come-up rate was 100 MPa per 15 s (or 6.7 MPa/s) and the release rate was 100 MPa per 9 s (or 11.1 MPa/s). Samples were pressure-treated at 500, 400, and 300 MPa (4°C) at 5 min intervals for up to 25 min. The initial temperature in the processing chamber was ca. 4°C and did not exceed a maximum of 35°C during the HPP treatment. Keeping the chamber temperature low (ca. 4°C) prevents compression heating induced thermal effects from interfering with HPP inactivation kinetic determination (Sheen et al., 2015). The chamber temperature was monitored by the built-in sensor (a T-type thermal couple device). The thermal sensor was immersed in the working chamber near food samples filled with the recirculation fluid.

### Gamma Radiation

A Lockheed Georgia Company (Marietta, GA, USA) self-contained ^137^Cs irradiator, with a dose rate of 0.065 kGy/min, was used for all exposures. The radiation source consisted of 23 individually sealed source pencils in an annular array. The 22.9 cm × 63.5 cm cylindrical sample chamber was located central to the array when placed in the operating position. Inoculated samples were placed vertically and centrally in the sample chamber, using a 4 mm thick polypropylene bucket, to ensure a good dose uniformity (DUR < 1.1:1.0). The temperature during irradiation (4°C) was monitored by thermocouple and maintained (4 or -20°C) by introduction of the gas phase from a liquid nitrogen source directly into the top of the sample chamber. The radiation doses were at 0.3 and 0.6 kGy increments at 4 or -20°C, respectively. The absorbed dose was verified using temperature tempered 5 mm alanine pellets that were then measured using a Bruker eScan EPR Analyzer (Bruker, Billerica, MA, USA).

### Exposure to Ultraviolet Light

A custom built UV-C apparatus (2 mW/cm^2^) (Sommers et al., 2010) was used to treat chicken purge inoculated with UPEC on stainless steel (304 L), High Density Polypropylene (HDPP) and High Density Polyethylene (HDPE) coupons (5 × 10 cm), and the foods themselves. Chicken purge was thawed in a refrigerator overnight and 0.5 ml of UPEC cocktail inoculated into 4.5 ml chicken exudates was then mixed by vortexing for 30 s. One hundred microliter of inoculated purge was placed on the coupons which were then spread to a 4 cm × 4 cm area using an inoculating loop. The coupons were placed in a refrigerator for 30 min and then placed on a cold pack (4°C) for UV-C exposure. The UV-C intensity exposure times were 0, 10, 20, 30, 40, 50, and 60 s for UV-C doses of 20, 40, 60, 80, 100, and 120 mJ/cm^2^.

For chicken meat and skin 4 × 4 cm sections (ca. 1 mm thick) of boneless skinless chicken breast were placed in sterile petri dishes and inoculated with 0.1 ml of chicken purge which was then spread onto the surface (4 cm × 4 cm) using an inoculating loop, and then incubated for 30 min in a refrigerator (4°C) prior to treatment with UV-C. The samples were placed on cold packs prior to UV-C treatment. The UV-C intensity exposure times were 0, 10, 20, 30, 40, 50, and 60 s for UV-C doses of 20, 40, 60, 80, 100, and 120 mJ/cm^2^.

UV-C intensity was monitored using a calibrated UVX Radiometer (UVP Inc., Upland, CA, USA). The temperature of the room was approximately 20°C during the exposure to UV-C, and the food temperature did not increase to more than 30°C at the end of the process as measured using an infrared thermometer.

### Recovery of the Surviving *E. coli*

The individual ground chicken samples were added to 90 ml of 0.1% PW and then stomached for 2 min (Model Bag Mixer 100W, Inter science Co., France). The coupons with 0.1 ml exudate were placed in stomacher bags with 9.9 ml SPW and hand massaged for 1 min. For recovery of UPEC 1.0 mL, after proper decimal dilutions, was placed on duplicate *E. coli*/coliform Petrifilm^TM^ (3M Microbiology Products Co., St. Paul, MN, USA). The films were maintained at room temperature for 4 h to allow the injured cells to recover (Hsu et al., 2014) and then incubated at 37°C for 24 h. Colonies (CFU) were enumerated for determination of log reduction and D_10_. Incubation for longer periods did not result in changes to the colony counts, an indicator of injured cell recovery.

### Statistical Analysis

The mean plate counts of the treated samples (N) were divided by the average control plate counts (No) to give a survivor ratio (N/No). The log_10_ (N/No) of the ratios was then used for determination of D_10_-values and other statistical analyses. D_10_-values were determined by the reciprocal of the slope following linear regression as determined by least squares analysis (Diehl, 1995). Each experiment (D_10_ determination) was conducted independently three times. A minimum of five time points were used for determination of D_10._ Statistical analysis functions of MS Excel (Microsoft Corp., Redmond, WA, USA) were used for routine calculations (D_10_ determination), descriptive statistics, analysis of variance (ANOVA, 95% confidence).

## Results and Discussion

### High Pressure Processing

**FIGURE 1:**
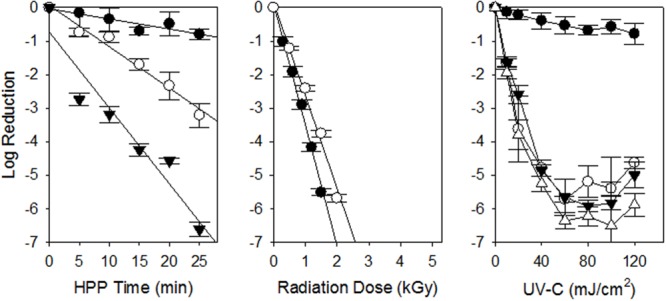
**Inactivation of uropathogenic *Escherichia coli* on chicken meat and chicken purge by non-thermal processing technologies.** HPP 300 (•), 400 (∘) and 500 (▼) MPa are shown as well as gamma radiation at 4 (•) and -20 (∘) °C. For UV-C inactivation of UPEC on chicken breast meat (•), and chicken exudates on SS (∘), HDPE (▼) and HDPP (Δ) are shown. Each experiment was conducted independently three times (*n* = 3). The standard error of the mean is shown as error bars. The linear regressions are shown as solid lines.

The HPP inactivation kinetics for the UPEC multi-isolate cocktail is shown in **Table 1** and **Figure 1**. As we have found previously for STEC the inactivation kinetics was first order in nature. The HPP D_10_ of the UPEC in refrigerated (4°C) ground chicken was ca. 30.6, 8.37, and 4.43 min at 300, 400, and 500 MPa, respectively. HPP treatment using 300 MPa was ineffective as a treatment as ca. 1 log was inactivated at that pressure. When we compare the results of this study with those from previous HPP studies for inactivation of STEC the results are similar. Sheen et al. (2015) found the mean HPP D_10_ (350 MPa) of 39 STEC isolates from illness outbreaks to be ca. 9.25 min while those from animals and environmental sources was ca. 10.4 min when suspended in 80% lean ground beef (350 MPa, 4°C). Hsu et al. (2014) found that 450 MPa (15 min, 4°C) inactivated 5.5–6.9 log of STEC in 77% lean ground beef while 350 MPa inactivated ca. 3.2–4.7 log. Jiang et al. (2015) was able to inactivate 3–4 log of STEC with HPP using multiple 1 min cycles at 400 MPa. Our results obtained using the UPEC were similar to those we have and others have obtained in the STEC suspended in ground beef.

**Table 1:** D_10_ values for uropathogenic *Escherichia coli* in ground chicken and chicken purge.

Technology	Parameter	D_10_ (SEM)
High pressure processing	300 MPa	30.6 (±0.12) min
	400 MPa	8.37 (±1.06) min
	500 MPa	4.4 (±0.1.2) min
Gamma radiation	4°C	0.28 (±0.01) kGy
	-20°C	0.36 (±0.01) kGy
Ultraviolet light (chicken purge)	Stainless steel	11.9 (±0.49) mJ/cm^2^
	HDPP	11.4 (±0.47) mJ/cm^2^
	HDPE	12.9 (±0.59) mJ/cm^2^

*D_10_ for HPP, GR, and ultraviolet light are shown with the standard error of the mean in parenthesis. Each experiment was conducted independently three times (*n* = 3). Each HPP D_10_ was significantly different than the others, as were the GR D_10_ (ANOVA, *p* < 0.05). There was no difference (*p* > 0.05) between the UV-C D_10_ for food contact surfaces.*

### Gamma Radiation

When the UPEC cocktail was suspended in ground chicken and treated with gamma radiation the GR D_10_ was ca 0.28 kGy at refrigeration (4°C) temperature (**Figure 1**, **Table 1**). These results are similar to those obtained by Sommers et al. (2015) which found the GR D_10_ of STEC associated with illness outbreaks to be ca. 0.27 kGy when suspended in refrigerated 80% lean ground beef. Sommers and Fan (2012) reviewed the studies for inactivation of *E. coli* O157:H7 in refrigerated ground beef in which the GR D_10_ ranged from 0.013 to 0.37 kGy. GR D_10_ for microorganisms irradiated in frozen foods are typically higher than that in refrigerated foods due to the limitation of indirect DNA damage through immobility of hydroxyl radicals produced by the radiolysis of water in the frozen state (Bruns and Maxcy, 1979; Taub et al., 1979). Lopez-Gonzalez et al. (1999) found the D_10_ for *E. coli* O157:H7 suspended in frozen beef (-15°C) beef to be 0.62 kGy. Thayer and Boyd (2001) found the GR D_10_ of *E. coli* O157:H7 in frozen ground beef (-20°C) to be 0.98 kGy. Black and Jaczynski (2006) obtained D_10_ of 0.33 and 0.35 kGy for *E. coli* O157:H7 in frozen (-20°C) ground beef and chicken, respectively. It appears the radiation doses needed to inactivate STEC in refrigerated and frozen meat and poultry products should also control the UPEC.

### Ultraviolet Light

In this study our objective was to calculate a UV-C D_10_ value for the UPEC suspended in chicken exudate on SS, HDPP, and HDPE surfaces. The UV-C D_10_ for UPEC is shown in **Table 1** and **Figure 1**. The D_10_ was calculated from the linear portion of the survival curve (0–60 mJ/cm^2^) and ranged from 11.4 to 12.9 mJ, cm^2^ (*p* > 0.05, ANOVA). As with previous studies complete inactivation of microorganisms with UV-C is difficult because of shadowing by particulates in purge. The D_10_ for UPEC in purge obtained were very similar to those obtained with STEC suspended in veal purge (Sommers et al., unpublished data), as well as other foodborne pathogens (Sommers et al., 2012; Sommers and Sheen, 2015). A relatively low UV-C dose of 100 mJ/cm^2^ should be able to inactivate ≥5 log of UPEC in chicken purge on food contact surfaces.

When we inoculated the UPEC onto skinless chicken meat we obtained ca. 0.6 (±0.19), respectively, which was significantly reduced from the untreated controls (*p* < 0.05) which is consistent with previous results from our group as well as other researchers (Stermer et al., 1987; Sumner et al., 1996; Sommers et al., 2010). When the UPEC were inoculated onto chicken skin we did not obtain a significant reduction, which is again consistent with results we have obtained using other foodborne pathogens on chicken skin (Stermer et al., 1987; Sumner et al., 1996; Sommers et al., 2010). The reduced inactivation of the UPEC on skin and meat surfaces is expected due to the surface topology and shielding of the UPEC from UV-C (Gardner and Shama, 2000).

## Conclusion

Our results indicate the HPP, GR, and UV-C inactivation kinetics of the UPEC are similar to our historical results for the STEC in meat and meat purge. The processing conditions used to control STEC should have similar effects on the UPEC.

## Disclaimer

Mention of trade names or commercial products in this publication is solely for the purpose of providing specific information and does not imply recommendation or endorsement by the U.S. Department of Agriculture. USDA is an equal opportunity provider and employer.

## Author Contributions

SS contributed to collection of high pressure processing data. OS was responsible for collection of UV-C data, CS was study director and designed the study, was responsible for data collection and analysis, and was responsible for manuscript completion.

## Conflict of Interest Statement

The authors declare that the research was conducted in the absence of any commercial or financial relationships that could be construed as a potential conflict of interest.

## References

[B1] Bao Y. Welk B. Reid G. Burton J. (2014). “Chapter 14-Role of the microbiome in the recurrent urinary tract infection,” in *Novel Insights into Urinary Tract Infections and their Management* ed. Matsumoto T. (London: Future Medicine Ltd.).

[B2] Bergeron C. Prussing C. Boerlin P. Daignult L. Reid-Smith R. Zhanel G. (2012). Chicken as a reservoir for extraintestinal pathogenic *Escherichia coli* in humans. *Canada. Emerg. Infect. Dis.* 18 415–421. 10.3201/eid1803.111099 22377351 PMC3309577

[B3] Black J. Jaczynski J. (2006). Temperature effect on inactivation kinetics of *Escherichia coli* O157:H7 by electron beam in ground beef, chicken breast meat, and trout fillets. *J. Food Sci.* 71 M221–M227. 10.1111/j.1750-3841.2006.00105.x

[B4] Bruns M. Maxcy R. (1979). Effect of irradiation temperature and drying on survival of highly radiation resistant bacteria in complex menstrua. *J. Food Sci.* 44 1743-1746.

[B5] Campus M. (2010). High pressure processing of meat, meat products and seafood. *Food Eng. Rev.* 2 256–273. 10.1007/s12393-010-9028-y

[B6] Cortes P. Blane V. Mora A. Dahbi G. Blanco J. Blanco M. (2010). Isolation and characterization of potentially pathogenic antimicrobial resistant *Escherichia coli* from chicken and pig farms in Spain. *Appl. Environ. Microbiol.* 76 2799–2805. 10.1128/AEM.02421-09 20228098 PMC2863447

[B7] Diehl J. (1995). *Safety of Irradiated Food* 2nd Edn (New York, NY: Marcel Dekker) 93–98.

[B8] Gardner D. Shama G. (2000). Modeling UV-induced inactivation of microorganisms on surfaces. *J. Food Prot.* 63 63–70. 10643771 10.4315/0362-028x-63.1.63

[B9] Hsu H.-Y. Sheen S. Sites J. Huang L. Wu J. (2014). Effect of high pressure treatment on the survival of Shiga toxin-producing *Escherichia coli* in strawberry puree. *Food Microbiol.* 40 25–30. 10.1016/j.fm.2013.11.019 24549194

[B10] Jakobsen L. Garneau P. Bruant G. Harel J. Olsen S. Porsbo L. (2012). Is *Escherichia coli* urinary tract infection a zoonosis? Proof of direct link with production animals and meat? Eur. *J. Clin. Microbiol. Infect. Dis.* 31 1121–1129. 10.1007/s10096-011-1417-5 22033854

[B11] Jakobsen L. Kurbasic A. Skjøt-Rasmussen L. Ejrnæs K. Porsbo L. Pedersen K. (2010a). *Escherichia coli* isolates from broiler chicken meat broiler chickens, pork, and pigs share phylogroups and antimicrobial resistance with community-dwelling humans and patients with urinary tract infection. *Foodborne Path. Dis.* 7 537–547. 10.1089/fpd.2009.0409 20039794

[B12] Jakobsen L. Spangholm D. Pedersen K. Jensen L. Emborg H.-D. Agersø Y. (2010b). Broiler chickens, broiler chicken meat, pigs, and pork as a source of ExPEC related virulence genes and resistance in *Escherichia coli* isolates from community dwelling humans and UTI patients. *J. Food Microbiol.* 142 264–272. 10.1016/j.ijfoodmicro.2010.06.025 20656368

[B13] Jiang Y. Scheinberg A. Senevirathne R. Cutter C. (2015). The efficacy of short and repeated high-pressure processing treatments on the reduction of non-O157:H7 Shiga-toxin producing *Escherichia coli* in ground beef patties. *Meat. Sci.* 102 22–26. 10.1016/j.meatsci.2014.12.001 25524823

[B14] Johnson J. Kuskowski M. Smith K. O’Bryan T. Tatini S. (2005). Antimicrobial-resistant and extraintestinal pathogenic *Escherichia coli* in retail foods. 2005. *J. Infect. Dis.* 191 1040–1049. 10.1086/428451 15747237

[B15] Krisko A. Radman M. (2010). Protein damage and death by radiation in *Escherichia coli* and *Deinococcus radiodurans*. *Proc. Natl. Acad. Sci. U.S.A.* 107 14373–14377. 10.1073/pnas.1009312107 20660760 PMC2922536

[B16] Lopez-Gonzalez V. Murano P. Brennan R. Murano E. (1999). Influence of various commercial packaging conditions on survival of *Escherichia coli* O157:H7 to irradiation by electron beam versus gamma rays. *J. Food Prot.* 62 10–15. 9921821 10.4315/0362-028x-62.1.10

[B17] Markland S. LeStange K. Sharma M. Kniel K. (2015). Old friends in new places: Exploring the role of extraintestinal *E. coli* in intestinal disease and foodborne illness. *Zoonoses Public Health* 62 491–496. 10.1111/zph.12194 25917531

[B18] Minardi D. d’Anzeo G. Cantoro D. Conti A. Muzzonigro G. (2011). Urinary tract infections in women: etiology and treatment options. *Int. J. Gen. Med.* 4 333–343. 10.2147/IJGM.S11767 21674026 PMC3108201

[B19] Mitchell M. Johnson J. Johnston B. Curtiss R. Mellata M. (2015). Zoonotic potential of *Escherichia coli* isolates from retail chicken meat products and eggs. *Appl. Environ. Microbiol.* 81 1177–1187. 10.1128/AEM.03524-14 25480753 PMC4292506

[B20] Mora A. Viso S. Lopez C. Pilar Alonso M. Garcıa-Garrote F. Dabhi G. (2013). Poultry as reservoir for extraintestinal pathogenic *Escherichia coli* O45:K1:H7-B2-ST95 in humans. *Vet. Microbiol.* 167 506–512. 10.1016/j.vetmic.2013.08.007 24008093

[B21] Moreno E. Andreu A. Pigrau C. Kuskowski M. Johnson J. Prats G. (2008). Relationship between *Escherichia coli* strains causing acute cystitis in women and the fecal *E.coli* population of the host. *J. Clin. Microbiol.* 46 2529–2534. 10.1128/JCM.00813-08 18495863 PMC2519474

[B22] National Advisory Committee on Microbiological Criteria for Food [Nacmcf]. (2006). Requisite scientific parameters for establishing the equivalence of alternative methods of pasteurization. *J. Food Prot.* 69 1190–1216. 16715826 10.4315/0362-028x-69.5.1190

[B23] Nordstom L. Liu P. Price L. (2013). Foodborne urinary tract infections: a new paradigm for antimicrobial-resistant foodborne illness. *Front. Microbiol.* 4:29 10.3389/fmicb.2013.00029 PMC3589730 23508293

[B24] Rastogi R. Kumar A. Tyagi M. Sinha R. (2010). Molecular mechanisms of ultraviolet radiation-induced DNA damage and repair. *J. Nucleic Acids* 2010 592980 10.4061/2010/592980 PMC3010660 21209706

[B25] Salvage B. (2014). *Strengthening Meat Safety. MeatPoultry.com: The Business Journal for Meat and Poultry Processors*. Available at: http://www.meatpoultry.com/Writers/Bryan%20Salvage/Strengthening%20meat%20safety.aspx?cck=1. [accessed 20 August 2015].

[B26] Sheen S. Cassidy J. Scullen O. Sommers C. (2015). Inactivation of a genetically diverse set of Shiga toxin-producing *Escherichia coli* in ground beef using high pressure processing. *Food Microbiol.* 52 84–87. 10.1016/j.fm.2015.07.001 26338120

[B27] Simonin H. Duranton F. de Lamballerie M. (2012). New insights into the high-pressure processing of meat and meat products. *Comp. Rev. Food Sci. Food Safety* 11 285–306. 10.1111/j.1541-4337.2012.00184.x

[B28] Sommers C. Fan X. (2012). “Irradiation of ground beef and fresh produce,” in *Nonthermal Processing Technologies for Food* eds Zhang H. Q. Barbosa-Cánovas G. V. Bala Balasubramaniam V. M. Dunne C. P. Farkas D. F. Yuan J. T. C. (Hoboken, NJ: Wiley-Blackwell) 236–246.

[B29] Sommers C. Rakjowski K. Scullen O. Cassidy C. Fratamico P. Sheen S. (2015). Inactivation of Shiga Toxin-Producing *Escherichia coli* in lean ground beef by gamma irradiation. *Food Microbiol.* 49 231–234. 10.1016/j.fm.2015.02.013 25846936

[B30] Sommers C. Scullen B. Paoli G. Bhaduri S. (2012). Inactivation of Francisella tularensis Utah-112 on food and food contact surfaces by ultraviolet light. *J. Food Proc. Technol.* S11-002 1–4. 10.4172/2157-7110.S11-002

[B31] Sommers C. Sheen S. (2015). Inactivation of avirulent *Yersinia pestis* on food and food contact surfaces by ultraviolet light and freezing. *Food Microbiol.* 50 1–4. 10.1016/j.fm.2015.02.008 25998808

[B32] Sommers C. Sites J. Musgrove M. (2010). Ultraviolet light (254 nm) inactivation of pathogens on foods and stainless steel surfaces. *J. Food Safety* 30 70–79. 10.1111/j.1745-4565.2010.00220.x

[B33] Stermer R. Lasater-Smith M. Brasington C. (1987). Ultraviolet radiation-an effective bactericide for fresh meat. *J. Food Prot.* 50 108–111. 10.4315/0362-028X-50.2.108 30965406

[B34] Sumner S. S. Wallner-Pendleton E. Froning G. Stetson L. (1996). Inhibition of *Salmonella* typhimurium on agar medium and poultry skin by ultraviolet energy. *J. Food Prot.* 59 319–321. 10463453 10.4315/0362-028x-59.3.319

[B35] Taub I. Halliday J. Sevilla M. (1979). Chemical reactions in proteins irradiated at subfreezing temperatures. *Ad. Chem. Serol.* 180 109–140. 10.1021/ba-1979-0180.ch006

[B36] Thayer D. Boyd G. (2001). Effect of irradiation temperature on inactivation of *Escherichia coli* O157:H7 and *Staphylococcus aureus*. *J. Food Prot.* 64 1624–1626. 11601718 10.4315/0362-028x-64.10.1624

[B37] Vincent C. Boerlin P. Daignault D. Dozois C. Dozois C. Dutil L. (2010). Food reservoir for *Escherichia coli* causing urinary tract infections. *Emerg. Inf. Dis.* 16 88–94. 10.3201/eid1601.091118 PMC2874376 20031048

